# Serum Ceramide Kinase as a Biomarker of Cognitive Functions, and the Effect of Using Two Slimming Dietary Therapies in Obese Middle Aged Females

**DOI:** 10.3889/oamjms.2015.030

**Published:** 2015-02-26

**Authors:** Maha I. A. Moaty, Suzanne Fouad, Salwa M. El Shebini, Yusr M. I. Kazem, Nihad H. Ahmed, Magda S. Mohamed, Ahmed M. S. Hussein, Atiat M. Arafa, Laila M. Hanna, Salwa T. Tapozada

**Affiliations:** 1 *Nutrition and Food Science Department, National Research Centre, Dokki, Giza, Egypt*; 2 *Food Technology Department, National Research Centre, Dokki, Giza, Egypt (Affiliation ID: 60014618)*

**Keywords:** Cognitive function, ceramide kinase enzyme, obesity, dietary therapy, middle aged females

## Abstract

**AIM:**

Highlighting the impact of obesity on mental and cognitive functions using serum ceramide kinase enzyme concentration as a biomarker for cognitive evaluation in the middle aged females, and also targeting to control the obesity and simultaneously postponing the deterioration of the cognitive functions, by implementing two slimming dietary therapies each incorporating different functional ingredients known to boost cognition.

**SUBJECTS AND METHODS:**

Ninety six obese middle aged females, divided into two groups volunteered to follow a low caloric balanced diet combined with two bread supplements composed essentially of barley flour and wheat germ mixed with either 5% turmeric, group (A); or with 5% ginger, group (B) for 4 weeks, phase (1); to be followed by the hypocaloric diet alone for another 4 weeks, phase (2).

**RESULTS:**

By the end of phase (1), the biochemical analysis showed a positive response of the levels of C-peptide and modified homeostatic model assessment of insulin resistance; also increased levels of the serum ceramide kinase enzyme, coupled with improved cognitive functions tests. Improvement of the relevant metabolic profile, fasting blood glucose, blood pressure and the anthropometric measurements was detected.

**CONCLUSION:**

Using dietary therapy supported by special formulas which contain active ingredients succeeded in reducing weight and improving both the metabolic profile and the cognitive functions.

## Introduction

There is a direct relationship between middle age body weight and the risk of developing dementia later in life, with increased risk for the obese [1]. Dementia is a syndrome characterized by gradual decline in cognitive abilities and neuropsychiatric symptoms. Dementia can affect memory, language, attention, judgment, planning, behavior, mood and personality. Mild cognitive impairment does not significantly impair daily activities, but often represents an earlier stage of cognitive decline [2].

There is no cure for the cognitive decline and dementia; a cure may only be achieved by prevention. Dementia cases are potentially attributable to seven risk factors: diabetes, midlife hypertension, midlife obesity, depression, physical inactivity, smoking and cognitive inactivity [3]. Brookmeyer et al. [4] further estimated that 3 million cases could be prevented worldwide by reducing the incidence of these risk factors by 25%.

Insulin resistance and excess insulin resulting as a consequence of obesity may play a role in reducing beta amyloid (Aβ) clearance from the brain, increasing the risk of dementia and Alzheimer disease (AD). Adipose tissue produces proteins and hormones that are related to excess insulin and inflammation that in turn have effects on the brain. Obesity is also associated with increased risk for high blood pressure, diabetes, cardiovascular disease and cerebrovascular disease, which affect brain health and increase the risk of dementia. Reviews of studies in this area have concluded that high midlife cholesterol increases the risk of later developing dementia by around 2 times [5]. Inflammation is known to play a role in the pathological brain changes and brain cell death that cause dementia [6, 7]. Therefore, several studies have investigated whether inflammatory biomarkers as C- reactive protein are associated with increased risk of dementia, and the potential for anti-inflammatory drugs to reduce the risk. It has been suggested that the improved glucose tolerance observed in the presence of thiazolidinediones or statins is likely related to their anti-inflammatory properties. Thus, it can be considered that obesity corresponds to a sub-clinical inflammatory condition that promotes the production of pro-inflammatory factors involved in the pathogenesis of insulin resistance [8].

Ceramides and related molecules are critical agents involved in the pathogenesis of mild cognitive impairment (MCI) or early Alzheimer’s-type neurodegeneration disease states. They can be generated in liver, adipose tissue or brain; cause insulin resistance. They are cytotoxic; increase in the central nervous system (CNS) with various dementia-associated diseases, including AD. They are lipid soluble, and therefore likely to readily cross the blood-brain barrier [9]. In mammalian cells, cermide-1-phosphate (C1P) is produced via the ATP-dependent mechanism of converting ceramide to C1P by the enzyme; ceramide kinase (CERK). CERK was first described as a calcium-stimulated lipid kinase that co-purified with brain synaptic vesicles, and to date, CERK is the only identified mammalian enzyme known to produce C1P in cells [10]. Many studies have shown that C1P is important for membrane biology and for the regulation of membrane-bound proteins, and the CERK enzyme has appeared to be tightly regulated in order to control both ceramide levels and production of C1P [11].

Diet, exercise and other aspects of our daily interactions with the environment have the potential to alter our brain health and mental function. It is now known that particular nutrients influence cognition by acting on molecular systems or cellular processes that are vital for maintaining cognitive function. This raises the exciting possibility that dietary manipulations are a viable strategy for enhancing cognitive abilities and protecting the brain from damage, promoting repair and counteracting the effects of aging [12].

The aim of this work was to highlight the effect of obesity on cognitive functions, using the important related biomarkers to predict changes in cognitive functions, and to define better tools for diagnosis and follow up. In addition, two functional food in the form of dietary supplements were studied to end up with the most efficient in helping to control obesity and deterioration in cognitive functions in the middle aged obese women, as a preventive strategy to reduce the incidence of the full picture of dementia later in life.

## Materials, Methods and Subjects

### Raw Materials

Whole meal naked barley grains (giza 129) was purchased from The Ministry of Agriculture. Wheat germ was purchased from the North Cairo Mills Company, Egypt. Turmeric *(Curcuma longa)* and ginger *(Zingiber officinale)* were obtained from local herbal shop (Dokki, Egypt). Skimmed milk, tomato sauce, corn oil, baking powder and salt were purchased from the local market.

### Preparation and Evaluation of the Bread

Basic and modified formulas were prepared by mixing the barley flour with 5% turmeric powder (formula 1), or with 5% ginger powder (Formula 2), then with other ingredients according to table (1). 14.7 ml of dextrose solution (5.93%) and a suitable amount of water were added according to AOAC [13] to be formed as Syrian bread. These formulas were baked in a special oven at 200 °C for about 15 minutes. Weight, volume, Specific volume, diameter, thickness and spread ratio of the bread were recorded.

**Table 1:** Formula composition of Syrian bread (g/ 100g).

Raw materials	Formula 1	Formula 2
Naked barley	60	60
Wheat germ	15	15
Turmeric	5	-
Ginger	-	5
Skimmed milk	10	10
Tomato sauce	1.5	1.5
Corn oil	5	5
Baking powder	2	2
Salt	1.5	1.5

### Analytical Methods

Moisture, ash, fiber, protein and fat contents of two formulas of the Syrian bread were determined according to AOAC [13]. Individual elements (Ca, P, K, Na, Fe, Zn and Mg) in all samples were determined according to the method described by Chapman and Pratt [14]. Fatty acids, amino acids and polyphenols were determined using standard methods [15, 16].

### Organoleptic Tests

The two formulas of the Syrian bread were evaluated for color (20), flavor (20), taste (20), crispiness (20), appearance (20) and overall acceptability (100) according to the method described in AACC [17].

### Subjects

Ninety six obese Egyptian women shared as volunteers in this study which lasted for 8 weeks, divided into two phases of four weeks each. The patients were divided into two groups: group (A); 50 patients with a mean age of 46.04 ± 1.88 years and a mean BMI of 37.64 ± 1.11 kg/m^2^, and group (B); 46 patients with a mean age of 47.33 ± 2.23 years and a mean BMI of 34.83 ± 1.49 kg/m^2^. The protocol of the study was approved by the National Research Center Ethics Committee. In addition, an informed consent was obtained from each volunteer. During the first phase, group (A) followed a low caloric balanced diet (1000-1200 Kcalories/day), supplemented by naked barley flour mixed with 5% turmeric powder that was baked in the form of Syrian bread (Formula 1), two servings were consumed with breakfast (40 g) and one serving with dinner (20 g), instead of Baladi bread. Group (B) consumed another formula of the bread made from naked barley flour supplemented with 5% ginger powder (Formula 2), with the same instructions. Phase (2) lasted for 4 weeks in which the volunteers were following only the same low caloric balanced diet, where baladi bread replaced the Syrian bread supplying the same caloric content. The participants were monitored clinically, biochemically and cognitively throughout.

### Anthropometric Parameters and Blood Pressure Measurements

Blood pressure was measured while they were sitting quietly for 5 minutes using a mercury sphygmomanometer, where the mean of three readings were recorded. Relevant anthropometric measurements were recorded including height, weight, waist (minimal waist) and hip circumferences using standard method [18]. Body fat (BF) as a percent from the body weight was measured by using Geratherm Body Fitness (B-5010), Germany. Waist to hip ratio (WHR) as minimal waist to hip circumferences in cm, and body mass index (BMI) as weight in relation to height (weight in kg/height^2^ in meter) were calculated.

### Blood Sampling and Biochemical Analysis

Fasting blood samples (12 hours fasting) were drawn from the patients, before starting the regimen (basal sample), at the end of the first phase (mid sample) and lastly at the end of second phase (last sample). Blood samples were allowed to clot and the sera were separated. Fasting blood glucose (FBG) was determined on fresh sera using glucose oxidase method [19]; the remaining sera were stored at -70 C until used for further analysis. Serum total cholesterol (T.cholesterol), high density lipoprotein-cholesterol (HDL-C) and triglycerides (TG) were determined using; cholesterol proceed No 1010, Stanbio [20], HDL-C proceed No 0599 StanioLiquicolor [21] and triglycerides proceed No 2100 [22] (Enzymatic methods) respectively. Friedewald formula [23] was used to calculate low density lipoprotein-cholesterol (LDL-C); LDL-C = (Total Cholesterol) - (HDL-C) - (TG/5). Serum C-peptide was detected by enzyme Immunoassay Test Kit, catalogue no. E29-071, IMMUNOSPEC Corporation, Netherland [24]. According to Li et al. [25], insulin resistance was expressed by modified homeostasis model assessment- insulin resistance (M.HOMA-IR); M.HOMA-IR = 1.5 + FBG (mg/dl) × fasting C-peptide (ng/ml)/2.800, in which insulin was replaced by C-peptide, so as to be applied on diabetic patients using exogenous insulin. Ceramide kinase (CERK) enzyme concentration was detected by Human CERK ELISA kit, catalogue no. 201-12-3437, Sun Red Shanghai [26].

### Dietary Recalls

Collecting detailed data about nutritional habits and intake through 24hours- recall dietary history. Analysis of food items using World Food Dietary Assessment, (WFDAS), 1995, USA, University of California.

### Cognitive and Mental Evaluation

Mini Mental State Examination (MMSE) was performed for evaluation of mental and cognitive status. Sleep quality, and the number of sleeping hours and their pattern were evaluated. Exposure to sun: time, duration and clothing. A scoring system of 3 points scale was then put to present the degree of exposure and adequacy. General subjective life stresses, life pattern to evaluate general activity and history of exercising were recorded and put on the 3 points scale [27].

### Statistical Analysis

All values were expressed as mean± SE. Two tailed student’s t-test was used to compare the two groups. Correlation between the different parameters was tested by Pearson test. P values <0.05 were considered statistically significant. SPSS window software version 17.0 (SPSS Inc. Chicago, IL, USA, 2008) was used.

## Results

Table 2 summarizes the average of moisture, protein, fat, crude fiber and ash of the two formulas of the Syrian bread with either turmeric or ginger.

**Table 2:** Chemical composition of the two types of the Syrian bread supplements.

Bread samples	Moisture (%)	Protein (%)	Fat (%)	Fiber (%)	Ash (%)	T. CHO (%)
Formula 1 bread (1)	8.22 ± 0.22^b^	18.12 ± 0.65^b^	8.22 ± 0.29^b^	6.25 ± 0.13^a^	3.16 ± 0.19^a^	64.25 ± 0.74^a^
Formula 2	9.11 ± 0.35^a^	20.5 ± 0.52^a^	8.92 ± 0.41^a^	5.95 ± 0.09^b^	3.25 ± 0.11^b^	61.38 ± 0.96^b^
LSD at 0.05	0.82	1.013	0.026	0.22	0.086	1.032

T. CHO: total carbohydrates.

Data presented in Table 3 show the sensory evaluation of the two Syrian bread supplements as a function of replaced barley flour with turmeric or ginger. Regarding color, flavor, taste, crispiness, general appearance and overall acceptability, it could be noticed that significant differences between the two formulas.

**Table 3:** Hunter color parameters of the two types of the Syrian bread supplements.

Bread samples	Color (20)	Flavor (20)	Taste (20)	Crispness (20)	Appearance (20)	Overall acceptability (100)
Formula 1	18.2 ± 0.22^a^	18.17 ± 0.66^a^	17.5 ± 0.1^b^	17.22 ± 0.26^b^	17.2 ± 0.69^b^	90.29 ± 0.65^a^
Formula 2	16.98 ± 0.35^b^	16.82 ± 0.57^b^	18.3 ± 0.2^a^	18.42 ± 0.39^a^	18.62 ± 0.58^a^	90.14 ± 0.62^b^
LSD at .05	0.96	1.025	1.12	1.052	1.013	0.122

Table 4 shows the mineral contents of the raw ingredients and the two types of bread. Adding wheat germ and either of the turmeric or the ginger to the two supplements enriched its mineral contents and raised most of their values.

**Table 4:** Minerals level (mg/100g) in dry tested samples

Serial No.	Samples	P	K	Ca	Mg	Na	Fe	Zn
1	Barely	368.3	483.5	49.4	89.4	78.3	4.8	4.5
2	Wheat germ	1126.1	1012.	51.6	323.7	36.7	14.5	21.1
3	Turmeric	26.5	271.3	28.3	13.9	15.8	3.3	0.35
4	Ginger	223.2	1879.4	72.4	231.1	32.5	21.8	3.9
5	Formula (1)	637.1	533.7	48.3	146.0	55.8	8.1	8.7
6	Formula (2)	723.6	612.5	59.7	157.6	60.6	6.9	7.5

Formula 1 (5 %) turmeric; Formula 2 (5 %) ginger.

Table 5 shows the phenolic compounds of the raw ingredients and the two types of bread. The final results showed that the two types of Syrian bread showed nearly equal values.

**Table 5:** Total phenols contents of dry tested samples (mg/ 100g).

Serial No.	Sample	Total phenols (as tannins)
1	Barely	5796.98
2	Wheat germ	7530.57
3	Turmeric	5471.14
4	Ginger	7561.66
5	Formula (1)	5338.3
6	Formula (2)	5460.0

**Figure 1:**
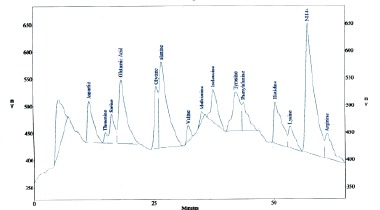
*Amino acids content (mg/g) in Formula (1): Aspartic (14.9), Thereonine (1.7), Serene (6.0), Glutamic (53.4), Glycine (4.6), Alanine (27.9), Valine (2.4), Methionine (2.5), Isoleucine (14.0), Tyrosine (31.7), Phenylalanine (15.6), Histidine (38.0), Lysine(10.5), Arginine (22.9) and Proline (3.9)*.

**Figure 2:**
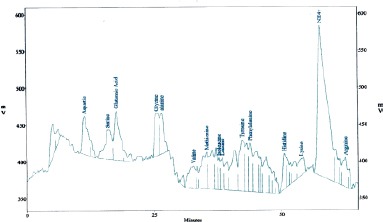
*Amino acids content (mg/g) in Formula (2): Aspartic (28.7), Thereonine (5.1), Serene (9.3), Glutamic (38.0), Glycine (4.7), Alanine (21.7), Valine (7.3), Methionine (20.1), Isoleucine (54.3), Tyrosine (70.5), Phenylalanine (18.5), Histidine (7.7), Lysine (2.3), Arginine (20.5) and Proline (10.3)*.

**Figure 3:**
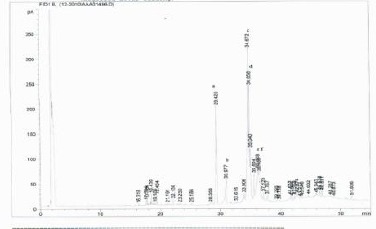
*Relative area (%) of fatty acid contents in Formula (1): (a) palmitic acid (C16:0, 14.8), (b) palmetoleic acid (C16:1, 4.4), (c) stearic acid (18:0, 27.24), (d) oleic acid (C18:1, 11.8), (e) linoleic acid (C18:2, 9.7) and (f) linolenic acid (C18:3, 6.2)*.

**Figure 4:**
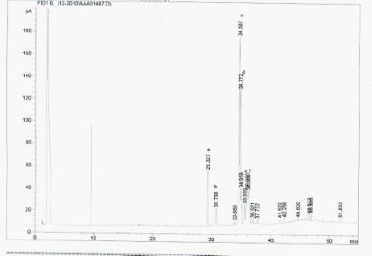
*Relative area (%) of fatty acid contents in Formula 2: (a) palmitic acid (C16:0, 8.7), (b) palmetoleic acid (C16:1, 3), (c) stearic acid (18:0, 0.5), (d) oleic acid (C18:1, 34.5), (e) linoleic acid (C18:2, 18.7) and (f) linolenic acid (C18:3, 10.6)*.

Fig. 1-4 shows the amino and fatty acids pattern of the two types of the Syrian bread. They were high in glutamine, histidine, tyrosine, alanine and arginine amino acids. Ginger bread (formula 2) showed higher content of the omega -3 fatty acids.

Table 6 shows the mean ± SE of the macro and micro nutrients of the habitual diet of the obese women and the three hypo-caloric regimens; with Baladi bread and with the two types of the Syrian bread. The habitual diet of the patients showed high rate of consumption of protein, fat and carbohydrate which reflected high caloric intake (123.52% of the RDA). The mean intake of vitamin A and D, and the mean mineral intake of calcium, iron and zinc were below the RDA. The three recommended regimens showed low caloric intake with sufficient protein, fat and carbohydrate, and better levels of the vitamins and the minerals.

**Table 6:** Mean ± SE & %RDA of macro and micronutrients of the habitual diet and the three types of dietary therapy among the obese women.

Nutrient intake	Habitual diet	Diet with Baladi Bread	Diet with Turmeric bread (formula 1)	Diet with Ginger bread (formula 2)	RDA

	Mean ± S E %RDS	Mean ± S E %RDS	Mean ± S E %RDS	Mean ± S E %RDS
Energy (kcal)	2717.53 ± 235.01 123.52%	974.25 ± 152.11 44.28%	904.28 ± 70.59 41.10%	901.02 ± 67.20 40.96%	2200

Protein (g)	91.23 ± 27.30 182.46%	52.63 ± 19.17 105.26%	52.23 ± 14.67 104.46%	53.05 ± 11.34 106.10%	50

Fat (g)	123.57 ± 37.08	30.17 ± 16.31	28.04 ± 13.20	27.18 ± 12.37	

Carbohydrates(g)	310.12 ± 60.96	123.05 ± 32.51	110.75 ± 28.71	111.05 ± 24.61	

Dietary fiber (g)	18.83 ± 9.97	29.19 ± 10.02	35.47 ± 8.91	35.60 ± 7.84	

Vit. A (µg)	598.12 ± 20.97 74.77%	771.54 ± 23.11 96.44%	775.31 ± 21.98 96.91%	778.51 ± 20.31 97.31%	800

Vit. D (µg)	2.16 ± 0.65 43.20%	3.61 ± 0.52 72.20%	3.78 ± 0.35 75.60%	3.75 ± 0.28 75.00%	5

Sodium (mg)	655.25 ± 50.30 131.05%	385.20 ± 30.25 77.04	320.89 ± 14.27 64.18%	309.78 ± 11.37 61.96%	500

Potassium (mg)	3231.78 ± 45.50 161.59%	1638.89 ± 60.35 81.94%	1645.82 ± 30.69 82.29%	1630.71 ± 26.30 81.54%	2000

Calcium (mg)	624.37 ± 70.61 62.44%	804.61 ± 36.25 80.46%	835.13 ± 26.91 83.51%	852.73 ± 27.32 85.27%	1000

Iron (mg)	7.35 ± 1.26 49.00%	11.20 ± 1.62 74.67%	11.30 ± 2.64 75.33%	11.26 ± 3.01 75.07%	15

Zinc (mg)	8.49 ± 2.08 70.75%	9.76 ± 1.54 81.33%	10.19 ± 1.84 84.92%	10.22 ± 1.37 85.17%	12

SFAs (g)	41.6023 ± 13.59	9.29 ± 2.39	9.03 ± 1.28	9.02 ± 1.39	

MUFAs(g)	37.3715 ± 11.75	10.46 ± 2.14	10.95 ± 1.89	10.88 ± 1.94	

PUFAs (g)	34.89 ± 11.69	7.77 ± 2.58	5.39 ± 1.94	5.36 ± 1.87	

Cholesterol (mg)	390.58 ± 44.06	89.20 ± 11.74	87.35 ± 4.58	87.65 ± 3.46	

SFAs; Saturated Fatty acids; MUFAs: Mono-Unsaturated Fatty acids; PUFA: Poly-Unsaturated Fatty acids.

Table 7 shows the base line results of the mini mental state examination (MMSE) which evaluate cognitive functions. The results show that 16.7 % has mild cognitive impairment, 72.9 % has good mental functions with no signs of cognitive impairment, while 10.4 % has excellent mental and cognitive functions. 33.4 % had very low hours of sleep, 60.4 % get moderate sufficient hours of sleep, while 6.2% get more than average hours of sleep. Sleep quality is low in 27.1%, moderate in 31.3% and high in 41.6%. What is clear that exposure to sun and general activity is low in general, while stress is very high in more than 50% of the sample. Moderate exercising is practiced by 56.3 % of the sample, low exercising in 31.2% and high exercising in 12.5%. The percent distribution of the sample as regard the MMSE, sleeping quality, general activity, exercising and exposure to sun were improved by the end of the intervention, contrary to the issue of stress which was still a challenge.

**Table 7:** Cognitive variables in percentage at the basal visit and after intervention

Variable	Score (1) % Basal	Score (1) % Last	Score (2) % Basal	Score (2) % Last	Score (3) % Basal	Score (3) % Last
MMSE	16.7	13.3	72.9	76.2	10.4	10.5
Sleeping hours	33.4	33.4	60.4	60.2	6.2	6.4
Sleeping quality	27.1	24.9	31.3	33.3	41.6	41.8
Exposure to sun	41.6	40.7	31.3	31.2	27.1	28.1
Stress	8.3	7.9	36.6	39.2	52.1	52.9
General activity	42.0	30.0	33.0	40.0	25.0	30.0
Exercising	31.2	28.0	56.3	58.5	12.5	13.5

MMSE: 22-25 =Score 1, 26-28=Score 2, 29= Score 3; Sleeping hours: 4-5 =Score 1, 6-8 = Score 2, 9-10 = Score3. Exposure to sun, Sleep quality, Stress, General activity, Exercising: 1=low 2=medium 3 =high

Table 8 shows the mean± SE of age, anthropometric and blood pressure measurements of the two groups at the start of the study (basal visit) and at the end of the two phases of regimen (mid and last visits). All the anthropometric measurements decreased significantly in both groups at p<0.05-0.01 at the mid and the last visits, except the BMI and %BF in group (A) which showed numerical decreases at the mid visit, while weight and BMI showed significant increases at the last visit. Both of systolic blood pressure in group (A), and diastolic blood pressure in group (B) showed significant decrease at the last visit only.

**Table 8:** Mean ± SE of age, anthropometric parameters and blood pressure among obese subjects at the three visits.

Parameters	Group (A) n= 50			Group (B) n= 46		

	Basal	Mid	Last	Basal	Mid	Last
Ages (year)	46.04 ± 1.88			47.33 ± 2.23		
Height (cm)	159.30 ± 1.17			157.50 ± 1.07		
Weight (kg)	94.79 ± 2.97	87.46 ± 2.37**^b^	86.53 ± 3.55	83.25 ± 3.43**^c^	86.47 ± 3.46*^a^	82.91 ± 3.64**^d^
BMI (kg/m^2^)	37.64 ± 1.11	34.38 ± 1.36	35.37 ± 0.88**^b^	34.83 ± 1.48	33.49 ± 1.48**^c^	33.52 ± 1.46**^d^
% BF	47.34 ± 0.92	46.32 ± 1.44	45.51 ± 1.42	43.02 ± 1.68	42.65 ± 1.63	41.40 ± 1.90
Waist (cm)	98.79 ± 2.16	91.97 ± 2.16**^a^	86.64 ± 2.29**^b^	91.89 ± 2.80	86.19 ± 2.56**^c^	83.44 ± 2.88**^d^
Abdominal II (cm)	122.00 ± 2.06	115.08 ± 2.67**^a^	110.54 ± 3.29**^b^	115.92 ± 2.76	109.75 ± 2.72**^c^	107.47 ± 3.19**^d^
Hip (cm)	122.53 ± 1.99	117.47 ± 2.85	113.66 ± 2.53**^b^	117.25 ± 1.92**^a^	112.23 ± 2.71**^c^	110.03 ± 2.97**^d^
WHR (cm/cm)	0.81 ± 0.014	0.78 ± 0.013**^a^	0.76 ± 0.018**^b^	0.78 ± 0.01	0.76 ± 0.01**^c^	0.75 ± 0.01
SBP (mmHg)	121.11 ± 2.96	121.11 ± 2.76	118.21 ± 3.08*^b^	124.71 ± 3.32	122.94 ± 1.70	123.21 ± 2.26
DBP (mmHg)	71.11 ± 2.31	71.11 ± 1.96	71.42 ± 2.53*^b^	74.12 ± 1.67	73.53 ± 1.70	72.31 ± 2.01*^d^

BMI: Body mass index; BF: Body fat; WHR: Waist to hip ratio; SBP: Systolic blood pressure; DBP: Diastolic blood pressure; a: Basal vs. Mid; b: Mid vs. Last in Group A; c: Basal vs. Mid; d: Mid vs. Last in Group B;

* P < 0.05;

** P < 0.01.

Table (9) shows the mean± SE of the different investigated biochemical parameters. At the end of phase (1), in spite of the slight elevation in the level of the FBG at the start of the study, as the diabetic participants were under medical treatment, yet the mean level of the FBG decreased significantly in both groups. The lipid profile disorders that were detected at the basal examination were improved significantly in both groups. Non- HDL-C is an important marker in such condition. C-peptide concentration and M.HOMA-IR values decreased significantly in both groups at p<0.05-0.01. The CERK enzyme concentration increased significantly in group (A), and numerically in group (B). At the last visit, the levels of these parameters increased with different ranges in both groups, while HDL-C and CERK concentrations decreased.

**Table 9:** Mean± SE of Biochemical parameters of the two groups at the basal and the end of the two phases of the dietary therapy.

Biochemical Parameters	Group A (no.=50)			Group B (no.=46)		

	Basal	Mid	Last	Basal	Mid	Last
FBG (mg/dl)	112.87 ± 4.66	99.53 ± 3.89**^a^	114.02 ± 4.84**^b^	119.58 ± 5.48	103.19 ± 4.77**^a^	106.38 ± 4.20
TG (mg/dl)	139.28 ± 13.10	88.86 ± 8.27**^a^	118.69 ± 7.91**^b^	135.24 ± 9.30	103.87 ± 7.37**^a^	102.09 ± 5.41
VLDL-C (mg/dl)	27.86 ± 2.62	17.77 ± 1.65**^a^	23.74 ± 1.58**^b^	27.05 ± 1.86	20.77 ± 1.47**^a^	20.42 ± 1.08
T. cholest.(mg/dl)	243.54 ± 10.18	185.32 ± 6.62**^a^	205.09 ± 10.53	217.01 ± 9.05	180.57 ± 7.31**^a^	183.72 ± 5.99
LDL-C (mg/dl)	167.74 ± 10.16	113.21 ± 7.55**^a^	129.37 ± 10.44	143.56 ± 8.75	107.99 ± 7.87**^a^	112.05 ± 5.67
HDL-C (mg/dl)	47.93 ± 1.53	54.33 ± 1.74**^a^	51.98 ± 1.53	46.39 ± 1.38	51.81 ± 1.29**^a^	51.25 ± 1.64
Non-HDL (mg/dl)	195.61 ± 10.84	130.98 ± 7.16**^a^	153.11 ± 10.88*^b^	170.61 ± 8.93	128.76 ± 7.87**^a^	132.47 ± 6.07
Risk factor	5.17 ± 0.29	3.47 ± 0.18**^a^	3.99 ± 0.26*^b^	4.73 ± 0.22	3.54±0.18**^a^	3.63 ± 0.15
C-peptide (ng/ml)	4.93 ± 0.97	3.72 ± 0.85*^a^	4.05 ± 0.91*^b^	5.07 ± 0.74	3.59 ± 0.47**^a^	3.72 ± 0.56
M. HOMA-IR	1.69 ± 0.04	1.63 ± 0.03**^a^	1.67 ± 0.04**^b^	1.72 ± 0.04	1.64 ± 0.02**^a^	1.65 ± 0.03
CERK (ng/ml)	17.27 ± 3.80	18.02 ± 3.95*^a^	17.67 ± 4.56**^b^	15.51 ± 3.50	16.34 ± 3.37	14.62 ± 3.09

FBG: Fasting blood glucose; TG: Triglycerides; VLDL-C: Very low density lipoprotein cholesterol; T. cholest.: Total cholesterol; LDL-C: Low density lipoprotein cholesterol; HDL-C: High density lipoprotein cholesterol; M.HOMA-IR: Modified homeostatic model assessment of insulin resistance; CERK: Ceramide kinase; a: Basal vs. Mid; b: Mid vs. Last;

* P<0.05;

** P<0.01.

## Discussion

The data obtained in this study concerning chemical composition, color attributes and sensory evaluation of Syrian bread supplements showed that the protein, fat, ash and total carbohydrate of Syrian bread produced from barley flour with ginger (formula 2) were higher than Syrian bread produced from barley flour with turmeric (formula1); however the fiber content of formula 1 was higher than that of formula 2. Therefore, it could be concluded that barley flour

could be mixed with either turmeric or ginger without drastic effect on the technological quality and sensory properties of Syrian bread. Moreover, higher nutritive values of this Syrian bread are achieved. Supplementation with skimmed milk, corn oil, turmeric and ginger improved their contents of protein, fat, fiber, ash, β glucan and minerals. Such findings are in-agreement with several previous studies [28-31].

Nearly similar significant decreases in the mean levels of most of the anthropometric measurements of the two groups were reported at p<0.05 & 0.01. After omitting the supplements, most of the anthropometric measurements still decreased in both groups, yet significant increase was reported in group (A) only in the mean weight and BMI, this might be attributed to an increase in the muscle mass, as a decrease in the mean value of the %BF was reported.

Small variations in the recorded values of the blood pressure were observed. Most of these values were found within the normal ranges as most of the hypertensive patients were under medical treatment; however both significant and numerical decreases in some of these values were reported in the different phases. The low caloric value and the bioactive components of the prescribed dietary therapy like fiber, phenolic compounds, omega-3 fatty acids and potassium might be the contributing factors that influenced the body weight and blood pressure.

The data of this study demonstrated an important healthy beneficial effect of both types of bread on the biochemical parameters which was high in its value when compared to their effect on the anthropometric measurements. At the last visit, the mean serum concentrations of the FBG, TG and the non-HDL-C significantly increased in group (A) compared to the values obtained at the mid visit, where a further increase in the mean level of the TG (33.6%) was reported. Furthermore, the mean concentration of the HDL-C decreased numerically in the same group. In group (B) the mean concentration of the FBG and most of lipid parameters increased numerically. Our data are in agreement with Shatwan et al. [32] who stated that consuming diets containing either cinnamon, barley, or their combinations regulate blood glucose, lipid profile, and adipose tissue hormones in type 2 diabetic rats. The mechanism that explains the link between diabetes and cognitive impairment involve vascular, metabolic, and inflammatory/oxidative processes [33]. In addition, in this study improving of the C-peptide concentration, the M.HOMA-IR values and the cognitive functions after the supplemented dietary intervention might add a further support to this suggested link.

Ceramides are lipid signaling molecules that cause cytotoxicity and cell death mediated by insulin resistance, inflammation, and endoplasmic reticulum (ER) stress. Concomitantly, insulin resistance dysregulates lipid metabolism, which promotes the complications of the ceramide accumulation. The toxic ceramides generated external to the CNS, as the liver, are released into the peripheral blood, and are subsequently transmitted across the blood-brain barrier to the brain where they induce brain insulin resistance, inflammation, and cell death [9].

Ceramide is involved in the generation of insulin resistance, so it is important to prevent its elevation in human body via increasing the activity of CERK enzyme that converts the ceramide to C1P via its phosphorylation [34]. C1P is a sphingolipid metabolite that has been implicated in membrane fusion of brain synaptic vesicles and neutrophil phagolysosome formation. C1P is a key regulator of cell growth and survival, it stimulates DNA synthesis and cell division, and it is a potent inhibitor of apoptosis [35].

The data of this study showed improvement in the serum level of the enzyme CERK after using of the dietary supplements (mid visit), that improved the metabolic profiles including insulin resistance. In this context, the biochemical response was more sensitive than the oral tests in reflecting the improvement of the brain physiology. This is because the results of the oral tests were alike in both the mid and last visits of both phases. Many studies have shown that C1P is important for membrane biology and for the regulation of membrane-bound proteins, and that the CERK enzyme has appeared to be tightly regulated as it is pivotal in controlling both the ceramide level and the production of C1P [11].

In conclusion, the data obtained in this study give further support to the relation between the prevalence of obesity and its complication in the deterioration of the cognitive functions in middle aged women. In addition, using dietary therapy supported by special formulas which contain active ingredients succeeded in reducing weight and improving both the metabolic profile and the cognitive functions thus alleviating the burden of cognitive impairment and dementia in the future.
